# MRE11 and UBR5 Co-Operate to Suppress RNF168-Mediated Fusion of Dysfunctional Telomeres

**DOI:** 10.3389/fonc.2021.772233

**Published:** 2021-11-22

**Authors:** Yongjian Tang, Joydeep Mukherjee, Russell O. Pieper

**Affiliations:** ^1^ Department of Neurosurgery, Xiangya Hospital, Central South University, Changsha, China; ^2^ Department of Neurological Surgery, University of California-San Francisco, San Francisco, CA, United States

**Keywords:** telomere, MRE11, UBR5, fusion, glioma

## Abstract

TRF2 is part of the shelterin complex that hides telomeric DNA ends and prevents the activation of the cNHEJ pathway that can lead to chromosomal fusion. TRF2, however, also actively suppresses the cNHEJ pathway by recruiting two proteins, MRE11 and UBR5. MRE11 binds BRCC3, which in turn deubiquitinates γH2AX deposited at exposed telomeric DNA ends and limits RNF168 recruitment to the telomere. UBR5, in contrast directly ubiquitinates and destroys RNF168. The loss of telomeric RNF168 in turn blocks the subsequent recruitment of 53BP1 and prevents the cNHEJ-mediated fusion of chromosomes with exposed telomeric DNA ends. Although MRE11 and UBR5 are both involved in the control of telomeric RNF168 levels and the chromosome fusion process, their relative contributions have not been directly addressed. To do so we genetically suppressed MRE11 and UBR5 alone or in combination in glioma cell lines which we previously showed contained dysfunctional telomeres that were dependent on TRF2 for suppression of telomeric fusion and monitored the effects on events associated with telomere fusion. We here show that while suppression of either MRE11 or UBR5 alone had minimal effects on RNF168 telomeric accumulation, 53BP1 recruitment, and telomeric fusion, their combined suppression led to significant increases in RNF168 and 53BP1 telomeric recruitment and telomeric fusion and eventually cell death, all of which were reversible by suppression of RNF168 itself. These results show that MRE11 and UBR5 co-operate to suppress fusion at dysfunctional telomeres.

## Introduction

Human telomeres are DNA-protein complexes present at the end of chromosomes. The DNA component of telomeres consists of double stranded 5’-TTAGGG-3’ DNA repeats and a single stranded G-rich 3’ overhang (1). The DNA repeats fold back on themselves to form a T-loop structure while the 3’overhang additionally inserts into the duplex repeats to form a D-loop that hides the terminal DNA sequence (2). Both T- and D-loops are stabilized by the protein component of the telomere known as the shelterin cap. The shelterin cap is a 6-protein structure that interacts with telomeric DNA, prevents exposure of DNA ends, and limits DNA repair pathways that would otherwise lead to telomeric DNA fusion, genomic instability, and cell death (3, 4).

**Figure 1:**
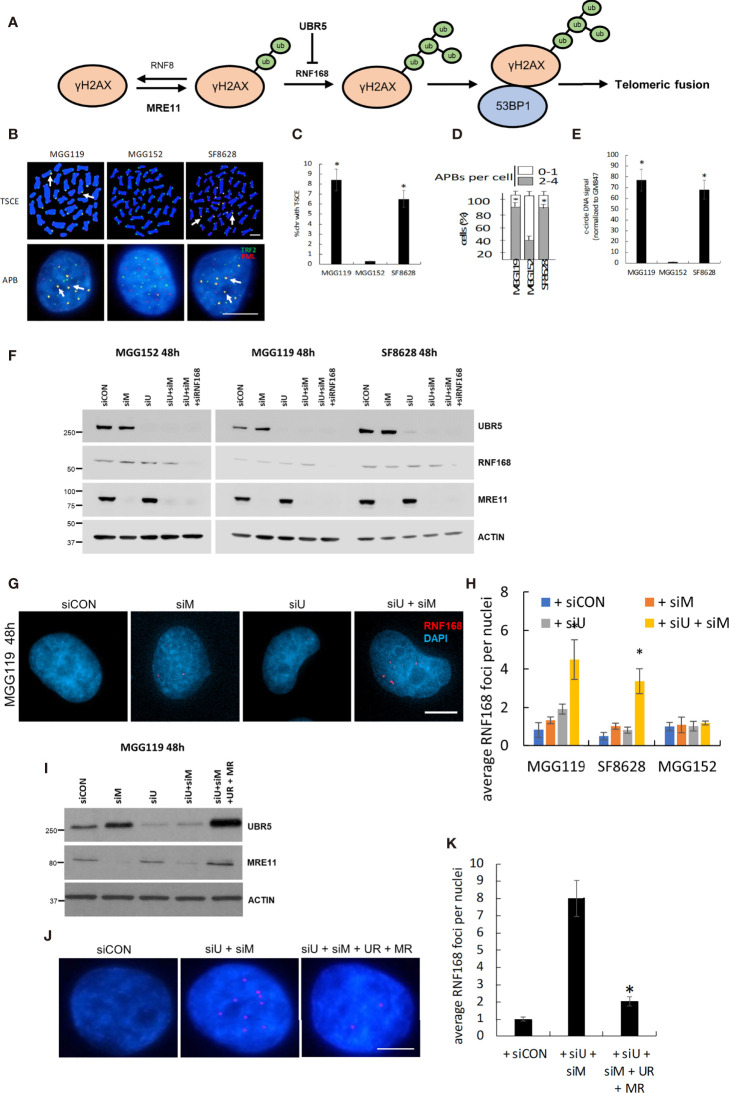
**(A)** Schematic of the basis for MRE11 and UBR5 mediated chromosome fusion in ALT-dependent cells. **(B)** Representative images and quantitation of T-SCE (CO-FISH,** C**), APBs **(D)**, and c-circle DNA **(E)** in MGG119, MGG152 and SF8628 cells. **(F)** Western blot verification of MRE11, UBR5, RNF168, and β-actin protein levels in MGG119, MGG152 and SF8628 cells 48hr after transfection with scrambled siRNA or pooled target siRNAs. **(G)** Representative images of MGG119 incubated with the indicated siRNAs for 48 hours and then immunostained with RNF168 antibody (red). **(H)** The average RNF168 foci number in each treatment of MGG119, MGG152 and SF8628 cells were counted, a minimum of 100 nuclei were assessed. **(I)** Western blot verification of MRE11, UBR5 and β-actin protein levels in MGG119 cells 48hr after transfection with scrambled siRNA, siRNAs targeting MRE11 and UBR5, or siRNAs targeting MRE11 and UBR5 followed by transfection with constructs encoding siRNA-resistant MRE11(MR) and UBR5(UR). **(J, K)** Representative image **(J)** and quantitation **(K)** of MGG119 incubated with the indicated siRNAs and siRNA-resistant MRE11(MR) and UBR5(UR) for 48 hours and then immunostained with RNF168 antibody (red). Mean ± SD of 3 independent experiments are shown. *P < 0.05.

Of the shelterin cap proteins, TRF2 plays a key role in suppressing initiation and propagation of a DNA damage response at telomeric ends. TRF2 contributes to the integrity of the T- and D-loops and in doing so limits activation of ATM and the DNA damage response (5). TRF2 also, however, prevents the propagation of a DNA damage response should one be initiated, by blocking the function of RNF168 (6, 7). RNF168 is a ubiquitin ligase whose recruitment to the telomere leads to ubiquitination of γH2AX, recruitment of 53BP1, and the cNHEJ-mediated fusion of chromosomes with exposed telomeric DNA (**Figure 1A**) (6, 8, 9). The iDDR domain found in the C-terminal hinge region of TRF2 recruits MRE11 and the deubiquitinase BRCC3, the latter which removes RNF8-mediated ubiquitin chains placed on γH2AX (9). Reduced levels of ubiquitinated γH2AX in turn limit RNF168 recruitment to the telomere. The same iDDR region also recruits UBR5, which ubiquitinates and destroys RNF168 (9, 10). As such TRF2-recruited MRE11 and UBR5 have been suggested to function in the same pathway to reduce levels of telomeric RNF168 and suppress the telomeric fusion that could occur upon exposure of telomeric DNA ends (9).

Although MRE11 and UBR5 have been suggested to work together in the same pathway to limit RNF168 accumulation and telomeric fusion, little experimental evidence exists to support this idea. In cells in which endogenous TRF2 was replaced with a chimeric TRF1/TRF2 iDDR molecule that limited the propagation, but not the initiation, of a telomeric DNA damage response, the suppression of either MRE11 or UBR5 significantly increased 53BP1 recruitment and chromosomal fusion (9), suggesting that MRE11 and UBR5 function independently to suppress fusion. Furthermore because both MRE11 and UBR5 were not suppressed in the same setting, the degree to which these two molecules work together to block fusion remains uncertain. To address the relative roles of MRE11 and UBR5 in the TRF2-mediated control of telomeric fusion, we genetically suppressed MRE11 and UBR5 alone or in combination in alternative lengthening of telomeres (ALT)-dependent glioma cell lines. ALT is a recombination-based mechanism in which telomeric DNA on one chromosomal arm is used as a template for DNA polymerase-mediated, telomerase-independent extension of telomeric DNA on a different arm (11). The mechanism is used by (5-10%) of many cancer sub-types, and nearly all lower-grade astrocytomas. We previously showed that these cells lines, by virtue of expression of mutant IDH1 and loss of ATRX, have dysfunctional telomeres that trigger a DNA damage response involving telomeric recruitment of both γH2AX and PARP1 (11, 12). The telomeres of these ALT-dependent cells, however, retain TRF2, which in turn is sufficient to prevent telomeric fusion. As such these cells make an optimal system in which to study the relative importance of MRE11 and UBR5 in the TRF2-mediated suppression of dysfunctional telomere fusion. Using this system, we here show that suppression of both MRE11 and UBR5 is required to trigger increases in RNF168 and 53BP1 recruitment, telomeric fusion, and cell death. These results therefore provide the first experimental evidence that that MRE11 and UBR5 work together in the same RNF168 pathway to suppress fusion at dysfunctional telomeres.

## Materials and Methods

### Tissue Culture and Cells

The MGG119 and MGG152 PDX xenograft cells were a gift from Dan Cahill (Massachusetts General, Boston, MA). These cells differ in ATRX status and ALT dependency: MGG119 are ATRX deficient and ALT dependent, whereas MGG152 are ATRX proficient and ALT independent (12, 13). SF8628 is an ATRX proficient, K27M mutated, and ALT dependent pediatric diffuse intrinsic pontine glioma (DIPG) cell line (14) obtained from the Brain Tumor Center (BTC) Tissue Bank at the University of California, San Francisco (UCSF). All cells were cultured in DMEM supplemented with 10% FBS and 1% penicillin/streptomycin (UCSF Cell Culture Facility) at 37°C in a 5% CO2 atmosphere. Cultures were confirmed mycoplasma negative (MycoSensor Mycoplasma Detection PCR Assay Kit) and used within three passages of thawing.

### Proliferation Assay

Proliferation of cells was determined using the MTT assay (Promega). The cells were plated in triplicate into 96-well tissue culture plates (1,000 cells/well). At specified time points, 15 μL of MTT dye solution were added to each well and incubated at 37°C for 4 h. A 100µl volume of the solubilization solution was added to terminate the reaction followed by measuring absorbance at 570nm using a microplate reader.

### Modulation of MRE11, UBR5, and RNF168 Expression

Cells were plated at 10^5^/mL in 6-well plates in DMEM media without antibiotics. Twenty-four hours later, the cells were transfected with an optimized amount of siRNA targeting human MRE11, UBR5, RNF168 (SMARTpool, Dharmacon) or nontargeting siRNAs as a control. After a 24 h exposure, cells were washed and grown an additional 24-72 h (48-96 h post exposure) before subsequent analysis. Cells were harvested for Western blot to verify target expression (>90%) 48h after exposure. Rescue experiments were performed using single siRNAs targeting MRE11 or UBR5 followed by transient transfection (48h) of an siRNA-resistant construct generated by introducing non-sense mutations at three sites in the coding sequence of Mre11 (5’ GGAAAUGAUACGUUUGUAA-3’) and UBR5 (5’- GAUUGUAGGUUACUUAGAA-3’) (Dharmacon ON-TARGETplus).

### Immunofluorescence (IF) Coupled With Fluorescence *In-Situ* Hybridization (FISH) Analysis

IF-FISH analysis was performed as described previously (12). In brief, paraformaldehyde-fixed cells were blocked at room temperature for 30 mins and then incubated with 53BP1 (1:200) primary antibodies for 18 hours at 4°C. Slides were incubated with fluorescent-tagged secondary antibodies (1:200; Alexa Fluor 488, 2 hours, Invitrogen). After washing, Cy3-labeled (CCCTAA)3 PNA oligonucleotide (Dako) were applied on the sections and were hybridized at room temperature overnight. The slides were washed, dehydrated in ethanol, air dried, and mounted on the following day (2). Zeiss LSM 510 microscope was used using a 63× objective and absolute amount of green, red, and yellow fluorescence localized to the nucleus in each cell was calculated using ZEN software (Zeiss). All FISH images presented contain amounts of telomeric foci within one SD of the mean for each given cell line

### Analysis of APBs and Telomeric Sister-Chromatid Exchange

ALT-associated PML nuclear bodies (APBs) were quantified using PML and TRF2 (1:200) primary antibodies as described previously (11). Co-localization of green and red fluorescence from the overlap of PML and TRF2 signal results in yellow foci was considered as APBs and was counted on >200 cells per group. Leading (TelC-Cy3-red) and lagging (TelG-FITC-green) strand probes (PNA Bio) as described previously (12) was used to quantify telomeric sister-chromatid exchange (T-SCE) by chromosome orientation fluorescence *in situ* hybridization (CO-FISH). Sister chromatid exchange results in overlapping of the leading and lagging strand probes generating yellow signal were considered T-SCE events. At least 50 cells per group were scanned and represented as the percentage of chromosomes exhibiting T-SCE. Negative controls were performed by omitting the peptide nucleic acid (PNA) probes, in which case nuclei examined exhibited no fluorescence.

### Analysis of Telomeric Fusion Using Telomeric FISH Probe

FISH was performed on metaphase spreads using a Cy3-labeled telomeric (CCCTAA)3 PNA oligonucleotide (Dako) followed by 4’,6-diamidino-2-phenylindole (DAPI) counterstaining as described previously (8). The percentage of metaphase spreads with 1 to >5 fused chromosomes (chromosomes with adjacent but not overlapping signal) was determined by counting 100 metaphase spreads in each cell group,

### Protein Extraction and Western Blot Analysis

Cells were washed with ice cold PBS lysed with RIPA lysis buffer (Life Technologies) adding 1× PhosStop and protease inhibitor cocktail (Roche). The protein concentration was measured by BCA protein assay (Bio-Rad). Equal amounts of protein (30 μg) were used for Western blot analysis as described (15). Primary antibodies against MRE11 (Novus Bio, 1:1000), UBR5 (Santa Cruz Biotechnology, 1:1000), RNF168 (Santa Cruz Biotechnology, 1:1000), b-actin (Cell Signaling Technology, 1:3000) were used. Antibody binding was detected using ECL reagents (Thermo Fisher Scientific).

### Statistical Analysis

All data were representative of at least 3 independent experiments and reported as means ± SD. Differences between two groups were analyzed by unpaired Student’s t test. Differences between multiple groups were analyzed by one-way analysis of variance (ANOVA) test with *post hoc* Tukey-Kramer multiple comparisons test. P < 0.05 was considered statistically significant.

## Results

To begin our studies, we first collected three glioma cell lines, MGG119, SF8628, and MGG152. MGG119 and SF8628 are adult and pediatric glioma cell lines, respectively and both display the characteristics of ALT-dependency as demonstrated increased telomeric sister-chromatid exchange (T-SCE) (**Figures 1B, C**), increased ALT-associated PML nuclear body (APBs) formation (**Figures 1B, D**) and presence of c-circle DNA (**Figure 1E**), relative to non-ALT MGG152 adult glioma cells**)** (11, 13, 14, 16, 17). We previously showed that siRNA-mediated suppression of MRE11 did not alter telomeric levels of RNF168, 53BP1, or telomeric fusion in ALT-dependent (MGG119) or ALT-independent (MGG152) cells when assessed at 24h post suppression (12). These results were consistent with those in the current study. Specifically, we noted that pooled siRNA targeting MRE11 led to a selective, >90% suppression of MRE11 protein levels (**Figure 1F**) 48h after exposure, but had no significant effect on RNF168 foci per DAPI-stained nucleus in control MGG119, SF8628, or MGG152 cells relative to scrambled siRNA controls (**Figures 1G, H**). Cells comparably incubated with a pooled siRNA targeting UBR5 also exhibited a selective, >90% suppression of UBR5 protein levels (**Figure 1**). Suppression of UBR5 levels, however, also had no significant effect on RNF168 foci per DAPI-stained nucleus in control MGG119, SF8628, or MGG152 cells relative to scrambled siRNA controls (**Figures 1G, H**). Combined siRNA-mediated suppression of MRE11 and UBR5 (**Figure 1F**), however, led to a significant increase in the number of RNF168 foci per DAPI-stained nucleus specifically in the cells with defective, exposed telomeric DNA (the ALT-dependent MGG119 and SF8628 cells), but not in the ALT-independent MGG152 cells (**Figures 1G, H**). Furthermore, introduction of constructs encoding siRNA-resistant forms of MRE11 and UBR5 in MGG119 cells with suppressed levels of these targets (**Figures 1I–K**), reversed the increase in the number of RNF168 foci per DAPI-stained nucleus. These results therefore show that suppression of MRE11 or UBR5 alone is insufficient to drive the process that leads to telomeric fusion.

**Figure 2:**
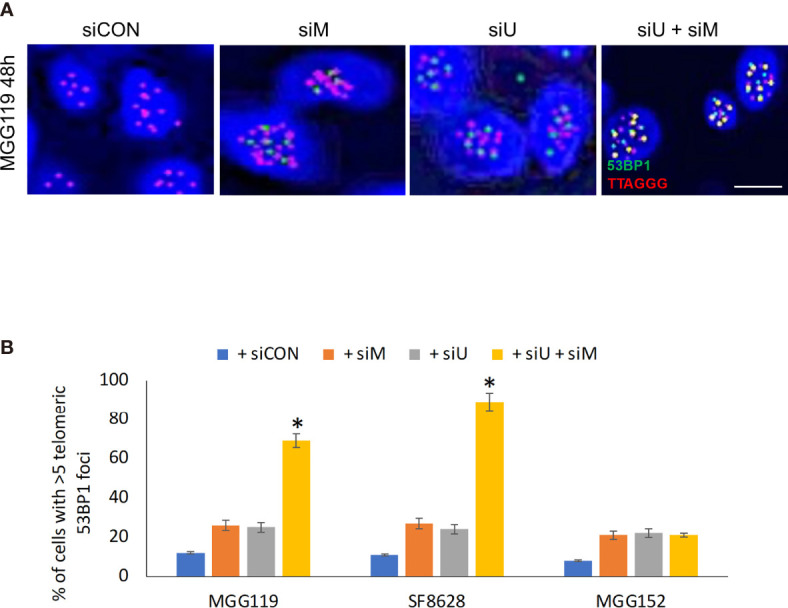
**(A)** Representative images of colocalized (yellow) immunofluorescence from a telomeric probe (TTAGGG, red) and a 53BP1 probe (green) in DAPI-stained MGG119 cells after 48 hours exposure to scrambled siRNA control (siCON) or pooled siRNA targeting MRE11 (siM), UBR5 (siU) or both. Scale bar, 10 μm. **(B)** Percentage of cells with >5 telomeric 53BP1 foci. Statistical significance relative to control (siCON) is indicated. Data are the means of triplicate samples ± SD derived from groups of >100 cells. *P < 0.05.

If MRE11 and UBR5 both work to limit accumulation of telomeric RNF168, we would also expect them to limit telomeric accumulation of 53BP1, a key component in cNHEJ-mediated fusion downstream of RNF168. Specifically, there was an equally low percentage of telomeres (identified by a TTAGGG-specific PNA probe) co-localized with 53BP1 (**Figures 2A, B**), in control MGG119, SF8628, or MGG152 cells or the same cells 48h after siRNA-mediated suppression of MRE11. siRNA-mediated suppression of UBR5 also did not significantly change the percentage of telomeric foci co-localized with 53BP1. Combined suppression of MRE11 and UBR5, however, led to a significant increase in percentage of telomeric foci co-localized with 53BP1 specifically in the ALT-dependent MGG119 and SF8628 cells at an early time point which is 48 hours after siRNA exposure, but not the ALT-independent MGG152 cells. These results therefore show that MRE11 and UBR5 work together to also limit accumulation of telomeric 53BP1.

**Figure 3:**
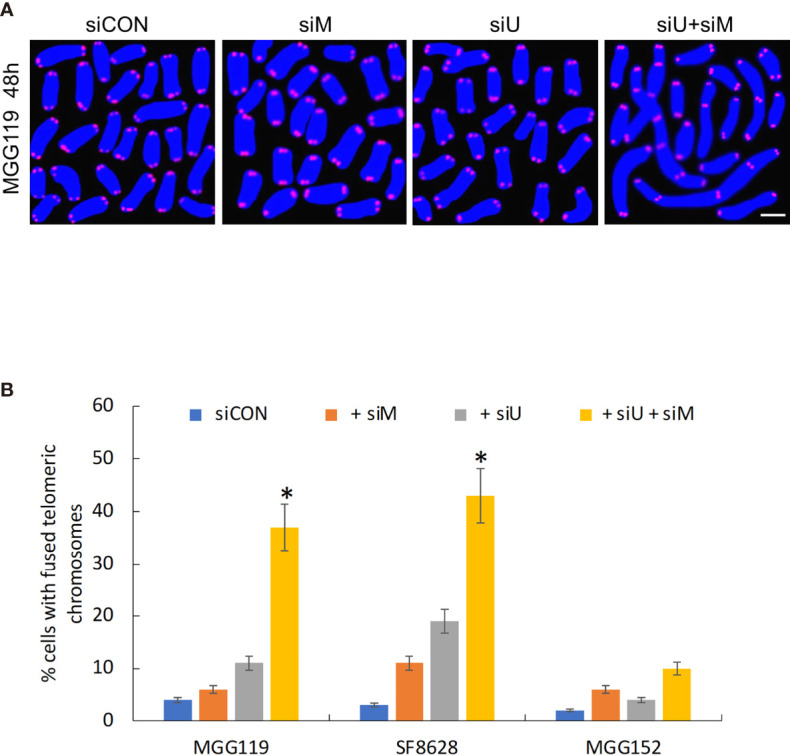
**(A)** Representative image of MGG119 cells after 48 hours exposure to scrambled siRNA control (siCON) or pooled siRNA targeting MRE11 (siM), UBR5 (siU) or both. Scale bar, 10 μm. DAPI is blue and telomere-specific probe is red. Scale bar, 10 μm. **(B)** Percentage of cells with >1 telomere-fused chromosome in metaphase spreads 48 hours after the indicated siRNA exposure. Significance relative to nontargeted (siCON) are indicated. *P < 0.05.

Finally, if MRE11 and UBR5 both work to limit accumulation of telomeric RNF168 and 53BP1, they should also limit the consequences of RNF168 and 53BP1 telomeric accumulation, namely telomeric fusion. After 48 hours of combined siRNA-mediated suppression of MRE11 and UBR5, there was a significant increase in percentage of cells with fused chromosomes specifically in the ALT dependent MGG119 and SF8628 cells but not in the ALT independent MGG152 cells (**Figures 3A, B**) relative to groups in which MRE11 or UBR5 were suppressed individually. These results therefore show that MRE11 and UBR5 both limit the RNF168-mediated pathway that drives fusion of dysfunctional telomeres.

**Figure 4:**
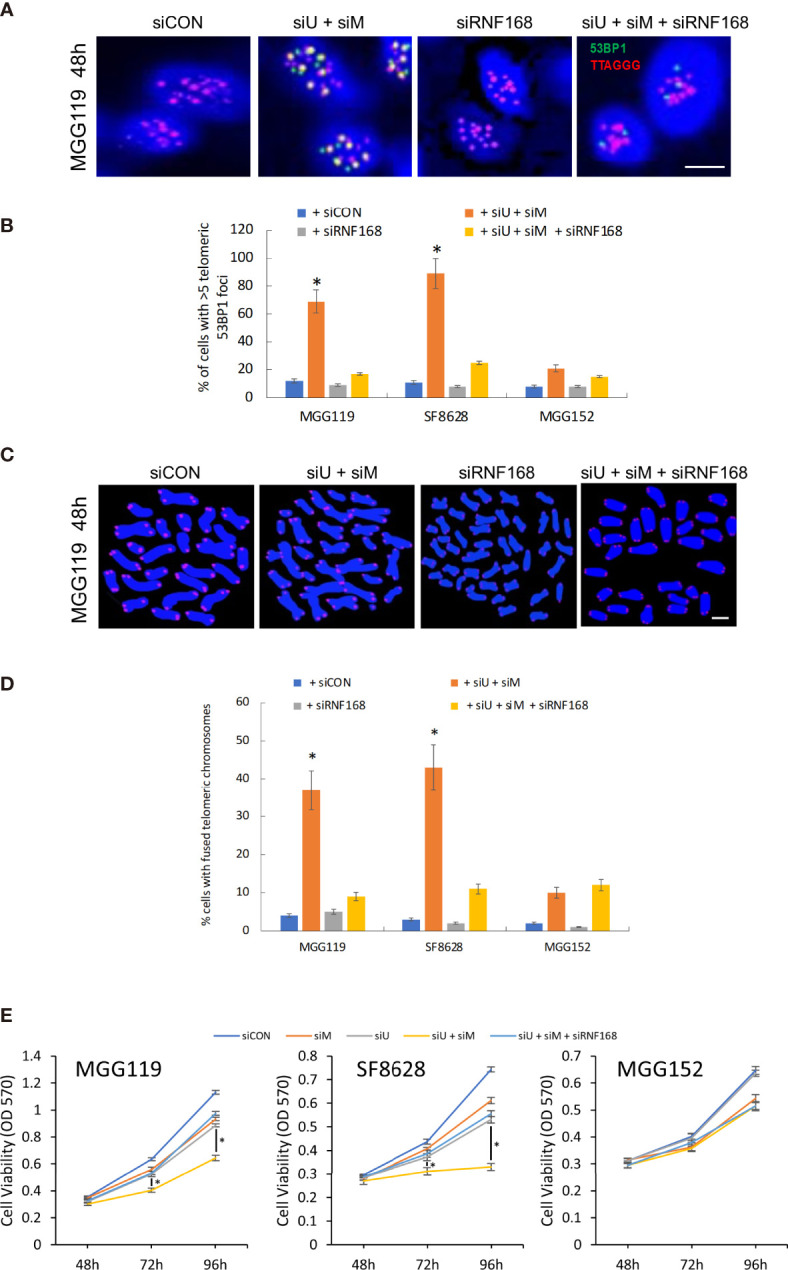
**(A)** Representative images of colocalized (yellow) immunofluorescence from a telomeric probe (TTAGGG, red) and a 53BP1 probe (green) in DAPI-stained MGG119 cells after 48 hours exposure to s scrambled siRNA control (siCON) or pooled siRNA targeting RNF168 (siRNF168), MRE11 and UBR5 (siU+siM) or adding siRNF168 (siU+siM+siRNF168). Scale bar, 10 μm. **(B)** Percentage of cells with >5 telomeric 53BP1 foci. Statistical significance relative to control (siCON) is indicated. Data are the means of triplicate samples ± SD derived from groups of >100 cells. *P < 0.05. **(C)** Representative image of MGG119 cells after 48 hours exposure to scrambled siRNA control (siCON) or pooled siRNA targeting RNF168 (siRNF168), MRE11 and UBR5 (siU+siM) or adding siRNF168 (siU+siM+siRNF168). DAPI is blue and telomere-specific probe is red. scale bar, 10 μm. **(D)** Percentage of cells with >1 telomere-fused chromosome in metaphase spreads 48 hours after the indicated siRNA exposure. Significance relative to nontargeted (siCON) are indicated. *P < 0.05 **(E)** Cell proliferation were measure by MTT assay at 48, 72 and 96 hours post siRNA exposure. Data are represented as the means ± SD of 3 independent experiments. *P < 0.05.

To further confirm that loss of MRE11 and UBR5 converge in the same pathway to increase levels of RNF168 and drive chromosomal fusion, we also performed converse experiments in which we determined if suppression of RNF168 itself blocked the stimulatory effects of MRE11/UBR5 loss on chromosomal fusion. For these studies MGG119, SF8628, and MGG152 cells were incubated with scrambled siRNA, siRNA targeting MRE11 and UBR5,siRNA targeting RNF168 and siRNA targeting MRE11,UBR5 and RNF168, after which effects on telomeric 53BP1 foci formation and chromosomal fusion were assessed. As shown in **Figures 4A, B**, while suppression of MRE11 and UBR5 led to the expected ALT cell-dependent increases in telomeric 53BP1 formation, and while suppression of RNF168 alone had no effect on 53BP1 accumulation, suppression of RNF168 in conjunction with suppression of MRE11 and UBR5 significantly reduced the induction of 53BP1 telomeric foci formation caused by suppression of MRE11 and UBR5. Consistent with these observations, the same siRNA-mediated suppression of RNF168 alone had no effect on chromosomal fusion but blocked chromosomal fusion induced by co-suppression of MRE11 and UBR5 (**Figures 4C, D**). As a whole these results show that MRE11 and UBR5 do not act in separate pathways to suppress RNF168-53BP1-mediated chromosomal fusion, but in fact work in the same pathway to limit levels of telomeric RNF168 and 53BP1-associated chromosomal fusion. Finally to determine if modulation of chromosomal fusion impacts viability, we performed MTT assay at various time points (48,72 and 96 hours) after siRNA mediated knockdown of MRE11 or UBR5, both MRE11 and UBR5, or of MRE11, UBR5 and RNF168. Combined knockdown of MRE11 and UBR5 significantly suppressed cell viability at 72 and 96 hours in ALT cells (MGG119 and SF8628) compared to non-ALT (MGG152) cells. Furthermore knocking down RNF168 rescued the decrease in viability observed from double knockdown of MRE1 and UBR5 (**Figure 4E**).

## Discussion

Cells that continually divide in the absence of telomerase gradually lose telomeric DNA, lose their ability to retain the shelterin cap, and eventually exhibit exposed telomeric DNA ends (18, 19). Such cells, which include those that have escaped from normal growth controls, are targets for telomeric fusion, which eliminates aberrantly proliferating cells from the population (20). Chromosomal fusion is therefore an important tumor-suppressive mechanism which in turn is carefully limited by members of the protective shelterin cap, and in particular TRF2. TRF2 was previously suggested to suppress the cNHEJ pathway that leads to fusion of exposed telomeric DNA by recruiting both MRE11 and UBR5, which were suggested to work together to suppress levels of telomeric RNF168 and 53BP1 (9). Experimental evidence in support of this co-operative action however was lacking, and published studies suggested that MRE11 and UBR5 in fact worked independently to suppress fusion (9, 12). The present studies, by studying fusion in a carefully controlled setting, provide the first direct experimental evidence that MRE11 and UBR5 do work together in the same RNF168 controlled pathway to prevent chromosomal fusion. This action could be considered to be co-operative in the sense that both MRE11 and UBR5 contribute in the same pathway to suppress fusion.

Previous studies by our lab and others showed that suppression of MRE11, the MRE11 partner BRCC3, or UBR5 alone were sufficient to induced chromosomal fusion in a manner comparable to that seen by combined suppression of both MRE11 and UBR5, or by loss of TRF2 itself (9, 12). On the surface these studies therefore suggest that MRE11 or UBR5 independently limit the entire RNF168-mediated fusion process. Our previous studies, however, showed that in addition to using BRCC3 to control telomeric accumulation of RNF168, MRE11 also uses BRCC3 in a delayed manner to control the stability of TRF2 itself (12). Therefore, at late time points (≥ 72h) following suppression of MRE11, loss of MRE11 alone caused telomeric fusion not because MRE11 alone limited the process, but because loss of MRE11 and TRF2 also likely eliminated the recruitment of both MRE11 and UBR5 (12). The analyses at earlier time points (48h) following MRE11 and UBR5 suppression as performed here, therefore allow a clearer picture of the relative contributions of MRE11 and UBR5 to the control of chromosome fusion, and clearly show that MRE11 and UBR5 play complementary roles in limiting telomeric DNA fusion.

Although the present studies link MRE11 and UBR5 in limiting RNF168-mediated fusion, some questions remain. First, it is unclear why large decreases in both MRE11 and UBR5 are required to unleash telomeric fusion. MRE11 and UBR5 both however have multiple functions in the cell, and as such the fusion control pathway may have evolved in manner that still allows for modulation of MRE11 and UBR5 levels independently without triggering cell death. Alternatively it may be possible that only small amounts of MRE11 and/or UBR5 may be sufficient to maintain γH2AX at levels below that need to trigger fusion. Additionally, the present data do not rule out the possibility that MRE11 and UBR5 function in the same pathway but in a redundant manner, such that either protein is sufficient to block the fusion process. The present data, however, clearly show that the RNF168-53BP1 cNHEJ fusion pathway is controlled by both MRE11 and UBR5. Recent studies have suggested that chromosome fusion and the mechanisms that control the process may be an important therapeutic target especially in ALT-dependent tumors (12). The present data aid in our understanding of this control process and further suggest that potential therapeutic approaches to trigger fusion in tumor cells with TRF2-containing dysfunctional telomeres (such as ALT-dependent tumors) will need to account for both MRE11 and UBR5 to be successful.

## Data Availability Statement

The original contributions presented in the study are included in the article/supplementary material. Further inquiries can be directed to the corresponding authors.

## Author Contributions

Study conception and design: YT, JM, and RP. Acquisition of data: JM and YT. Analysis and interpretation of data: YT, JM, and RP. Drafting of manuscript: RP, JM, and YT. All authors contributed to the article and approved the submitted version.

## Funding

Funding for this work was supported by R01 NS105087-01 and the loglio Project (RP).

## Conflict of Interest

The authors declare that the research was conducted in the absence of any commercial or financial relationships that could be construed as a potential conflict of interest.

## Publisher’s Note

All claims expressed in this article are solely those of the authors and do not necessarily represent those of their affiliated organizations, or those of the publisher, the editors and the reviewers. Any product that may be evaluated in this article, or claim that may be made by its manufacturer, is not guaranteed or endorsed by the publisher.
