# Expression of concern: MRE11 and UBR5 co-operate to suppress RNF168-mediated fusion of dysfunctional telomeres

**DOI:** 10.3389/fonc.2023.1308554

**Published:** 2023-12-07

**Authors:** Frontiers Editorial Office

“With this notice, Frontiers states its awareness of serious concerns surrounding the article “**MRE11 and UBR5 co-operate to suppress RNF168-mediated fusion of dysfunctional telomeres**” published on 2021 Nov 22. An investigation is currently being conducted by the **Office of Ethics and Compliance at the University of California San Francisco**, in accordance with applicable institutional policies and procedures. This expression of concern has been posted while Frontiers awaits the outcome of that investigation. It will be updated accordingly after that time.”

